# LIPID MAPS: update to databases and tools for the lipidomics community

**DOI:** 10.1093/nar/gkad896

**Published:** 2023-10-19

**Authors:** Matthew J Conroy, Robert M Andrews, Simon Andrews, Lauren Cockayne, Edward A Dennis, Eoin Fahy, Caroline Gaud, William J Griffiths, Geoff Jukes, Maksim Kolchin, Karla Mendivelso, Andrea F Lopez-Clavijo, Caroline Ready, Shankar Subramaniam, Valerie B O’Donnell

**Affiliations:** Systems Immunity Research Institute, School of Medicine, Cardiff University, Cardiff CF14 4XN, UK; Systems Immunity Research Institute, School of Medicine, Cardiff University, Cardiff CF14 4XN, UK; Babraham Institute, Babraham Research Campus, Cambridge CB22 3AT, UK; Systems Immunity Research Institute, School of Medicine, Cardiff University, Cardiff CF14 4XN, UK; Department of Pharmacology, Department of Chemistry and Biochemistry, University of California, San Diego, La Jolla, CA 92093-0601, USA; Department of Bioengineering, University of California, San Diego, 9500 Gilman Drive, La Jolla, CA 92037, USA; Babraham Institute, Babraham Research Campus, Cambridge CB22 3AT, UK; Swansea University Medical School, Singleton Park, Swansea SA2 8PP, Wales, UK; Systems Immunity Research Institute, School of Medicine, Cardiff University, Cardiff CF14 4XN, UK; Boehringer Ingelheim Espana SA, Carrer de Prat de la Riba, 50, 08174 Sant Cugat del Vallès, Barcelona, Spain; Systems Immunity Research Institute, School of Medicine, Cardiff University, Cardiff CF14 4XN, UK; Babraham Institute, Babraham Research Campus, Cambridge CB22 3AT, UK; Systems Immunity Research Institute, School of Medicine, Cardiff University, Cardiff CF14 4XN, UK; Department of Bioengineering, University of California, San Diego, 9500 Gilman Drive, La Jolla, CA 92037, USA; Systems Immunity Research Institute, School of Medicine, Cardiff University, Cardiff CF14 4XN, UK

## Abstract

LIPID MAPS (LIPID Metabolites and Pathways Strategy), www.lipidmaps.org, provides a systematic and standardized approach to organizing lipid structural and biochemical data. Founded 20 years ago, the LIPID MAPS nomenclature and classification has become the accepted community standard. LIPID MAPS provides databases for cataloging and identifying lipids at varying levels of characterization in addition to numerous software tools and educational resources, and became an ELIXIR-UK data resource in 2020. This paper describes the expansion of existing databases in LIPID MAPS, including richer metadata with literature provenance, taxonomic data and improved interoperability to facilitate FAIR compliance. A joint project funded by ELIXIR-UK, in collaboration with WikiPathways, curates and hosts pathway data, and annotates lipids in the context of their biochemical pathways. Updated features of the search infrastructure are described along with implementation of programmatic access via API and SPARQL. New lipid-specific databases have been developed and provision of lipidomics tools to the community has been updated. Training and engagement have been expanded with webinars, podcasts and an online training school.

## Graphical Abstract

**Graphical Abstract:**
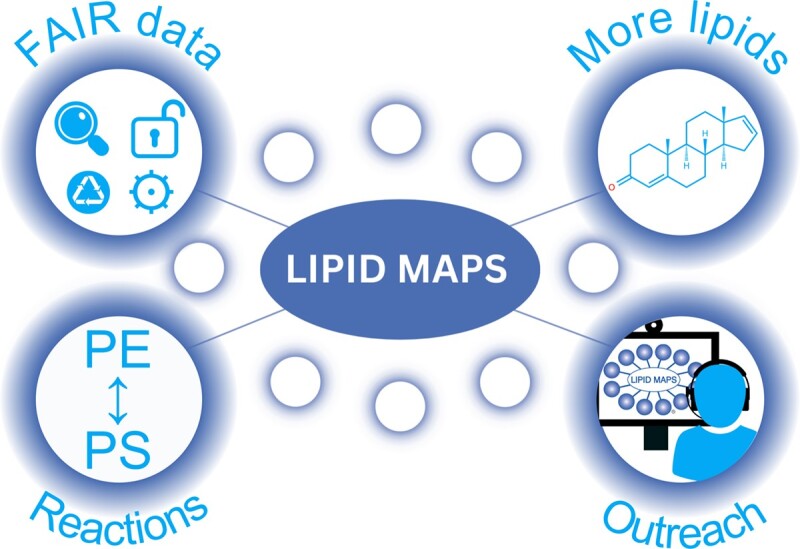

## Introduction

Lipids are biomolecules that play a vital role in living systems, serving not only as the building blocks of cell membranes and energy storage, but also as hormones, receptors and signaling molecules. They also play central roles in industrial applications including agrochemicals, pharmaceuticals and petrochemicals. Highlighting their importance, >60% of all metabolites detected and reported in studies deposited to the Metabolomics Workbench (1) are lipids. State of the art MS methods available nowadays allow researchers to profile thousands of lipid molecules in samples, leading to the generation of increasingly large datasets. These large scale studies can be categorized as lipidomics, a field which requires specialist informatics tools and resources, including both software and structured lipid databases, such as those provided by LIPID MAPS.

LIPID MAPS began as part of a large NIH grant to study lipids in 2003 led by Dennis with several co-investigators including Subramaniam who created the LIPID MAPS resource and spearheaded the bioinformatics efforts at the University of California, San Diego (UCSD), with the aim of advancing the field of lipidomics. The LIPID MAPS consortium established a classification, nomenclature and structural drawing system (2) that enabled the generation of databases including the LIPID MAPS structure database (LMSD) (3). In 2017, LIPID MAPS moved to the UK funded by a Wellcome Trust Biomedical Resources Grant led from Cardiff University, and run as a collaboration with UCSD, Babraham Institute and Swansea University. LIPID MAPS became an ELIXIR-UK data resource in 2020. From 2024 to 2029, it will be funded by the Medical Research Council (MRC) and also include the University of Edinburgh. Many developments have taken place during the 20 years of LIPID MAPS existence, and it has become the global standard for lipid classification, and the leading resource for databases, tools, protocols and standards in the lipid field. This paper describes recent developments to LIPID MAPS databases and website.

### Databases

#### Updates to LIPID MAPS Structure Database (LMSD)

LMSD is the primary database for fully characterized lipid chemical structures of biological relevance and was first introduced in this journal in 2007 (3). Since then, LMSD has increased in size to host >48 000 lipid structures. Lipids have been added from the scientific literature through manual curation, or incorporated from other specialist databases, such as in the case of flavonoids (4), carotenoids (5) and ascarosides (6). An interface for the scientific community studying lipids to submit new lipid structures as they identify and report them has been added. The LIPID MAPS classification system (2,3,7) has been expanded, with additional classes and subclasses added concomitant with the growth of the database. For instance, new classes and subclasses have been added for phosphatidylethanols, phosphatidylthreonines, ascarosides, betaine lipids and 1-deoxy ceramides. Additionally, some lipids have been reclassified, for instance fatty acid esters of hydroxy fatty acids (FAHFAs or fatty acid estolides) (8) have been removed from wax monoesters to form a new subclass. The wax monoester subclass has been divided such that esters where either the acid or alcohol moiety is fewer than five carbons are moved to a new subclass called Short Fatty Esters. Classification and nomenclature is overseen by the LIPID MAPS International Lipid Classification and Nomenclature Committee (ILCNC).

The LIPID MAPS system for assigning identifiers in LMSD (2,3) is unique in biochemical databases in that it encodes the classification ontology within the identifier. A consequence of this has been that up to now, reclassification of a lipid required a change of identifier. FAIR principles (9), however, require that identifiers need to be both globally unique and persistent. To address this, a change was implemented in how identifiers are assigned. Specifically, whilst identifiers continue to be indexed by identifiers.org (10) and will be assigned on the same basis as previously, reclassification no longer results in a new ID. As a result, new and legacy identifiers no longer should be considered to encode information about the classification ontology.

Individual lipids can include more than one functional group. Until now, however, lipids in LMSD were discoverable only in a single class. To address this, lipids can now be assigned to multiple relevant classifications, so that they are findable when browsing LMSD alongside structurally related compounds. Estrone 3-glucuronide (LMST05010011), for example, can be located either as a C18 steroid or as a glucuronide. Similarly, 11S-HETE, (LMFA03060003) is discoverable either as an eicosanoid, an unsaturated fatty acid or a hydroxy fatty acid. Where relevant, the shorthand nomenclature as defined by Leibisch *et al.* (11) is also included in the lipid record.

In 2020, LIPID MAPS began using the NCBI taxonomy (12) for annotation of curated lipids in LMSD. This provides source information about where the lipid was detected at the organism level. Work to back-populate taxonomy data for previously cataloged molecular species is ongoing, and so far approximately 18 000 entries in LMSD contain this information. FAIR principles request that data are associated with a detailed provenance, and to that end, references to the literature from which a lipid has been curated are now included in every new case, presented in a standardized format and linked to EuropePMC (13).

Historically, glycan groups within LMSD were depicted with a perspective representation of pyranose and furanose sugars in chair, or Haworth projections. While these may be visually preferable for biochemists, cheminformatics software are unable to interpret these depictions from a molfile (14). This results in inaccurate line representations (SMILES, InChI). To correct this, and to aid findability and interoperability, the molfiles of approximately 9500 lipids containing sugar groups have been recreated with the sugar in a planar representation and line representations recalculated. For visualization purposes however, the perspective representation continue to be displayed on the LMSD entry page.

Following this, mapping to ChEBI (15) and PubChem (16) was updated using the UniChem tool (17). Also utilizing UniChem mapping, links to the Protein Data Bank in Europe (18) have been added, where lipids are bound to macromolecular structures.

**Figure 1.**
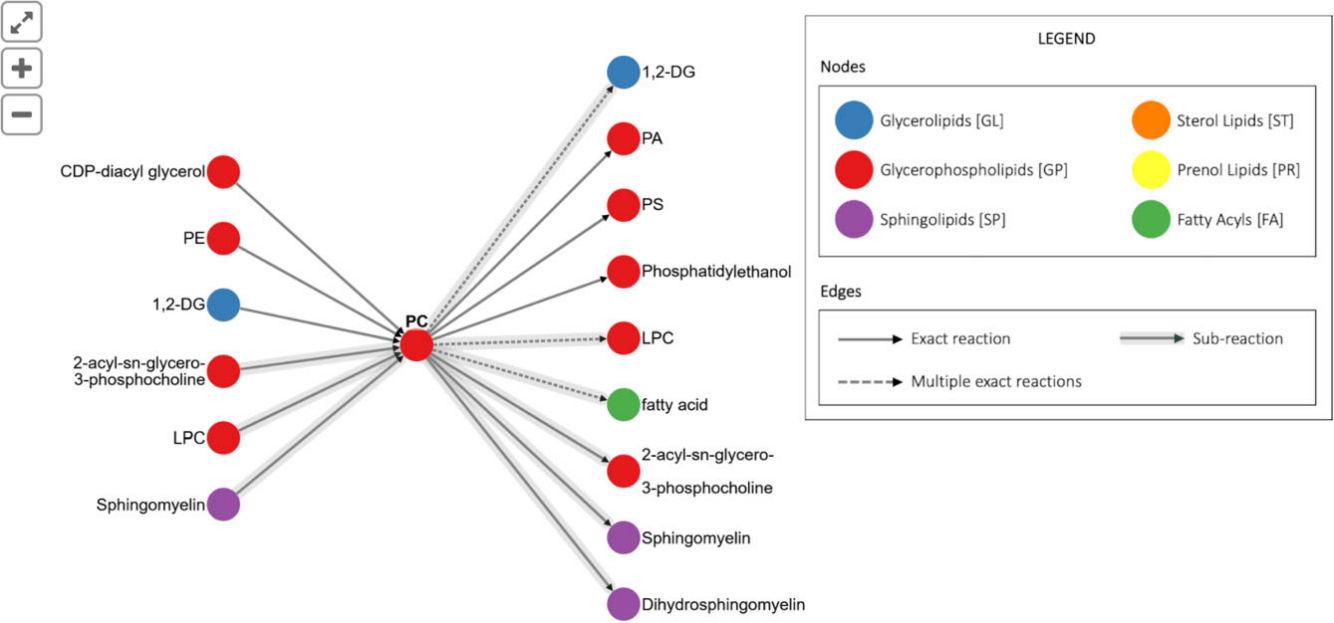
Example of biochemical reactions displayed on a lipid page in LMSD. Clicking on an arrow displays the details of a particular biochemical transformation.

Since 2022, LMSD has begun including biochemical reactions catalyzed by enzymes, as well as some non-enzymatic reactions. Here, individual pages for lipids show the reactions in which a lipid may take part. In this representation lipids are shown as nodes, and reactions as edges (Figure 1). Selecting an edge reveals specific details of the biochemical reaction and links to the source data describing that reaction. Reactions have been incorporated from LIPID MAPS-verified WikiPathways (19), Rhea (20), Reactome (21) and from expert biochemists via a community curation project funded in part by ELIXIR-UK, in collaboration with WikiPathways. In this project, experts provided details of pathways which were then added to WikiPathways to provide a visual representation. These were verified manually before incorporation into LIPID MAPS, where they are now embedded in a static form, linking back to LMSD entries. This work is ongoing and will continue as long as new lipids are added to LMSD, and as their reaction information becomes available. In addition to displaying reaction information on individual LMSD pages, all reactions are displayed together in the Reactions Explorer (www.lipidmaps.org/resources/tools/reactions) which allows navigation through this complex network. Filtering tools enable a user to quickly find relevant reactions for a lipid class of interest.

In many cases, the same reaction applies to many lipid molecular species (for example, phosphatidylethanolamine, PE, conversion to phosphatidic acid, PA, where there are many species differing only in fatty acyl composition). To accommodate this, the reactions database is constructed in such a way that only a generic PE → PA reaction needs to be added without specifying the fatty acyls. This cascades down such that the reaction is shown for all relevant lipids of the specific classes, maintaining the cognate radyl groups in the reaction. Ultimately, these data will be incorporated into BioPAN (22), a LIPID MAPS tool which performs pathway analysis.

#### Bulk searches

Searching lipidomics data to assign lipid names based on molecular mass using databases presents specific challenges. In particular, if only partial information on structures is provided, then fully annotated lipid names should not be used. To address this, LIPID MAPS has incorporated a shorthand annotation system which was originally published by Liebisch *et al.* to describe lipids at various levels of characterization (11,23). A quick guide to this nomenclature has been added at www.lipidmaps.org/shorthand_nomenclature to facilitate using this format. This is an evolving document as new lipid classes are added.

Mass spectrometry data often cannot distinguish regiochemistry such as *sn* position of radyl groups on (phospho)glycerolipids or double bond positions in acyl chains. It is, therefore, inappropriate to annotate full names of lipids in lipidomics data obtained without fragmentation. Instead, names should be assigned only at the ‘species level’, where the lipid type and sum composition (number of carbons and double bond equivalents) is defined. A new database, COMP_DB, has been developed containing over 60 000 lipids described at this ‘species’ level. This is searchable using a list of *m/z* values of various adducts rather than the neutral mass of the lipid and searches may be limited by selection of lipid category/class/sub-class, polarity or by instrument resolution level. The same interface can also be used to query LMSD. Once results are returned, users can drill down to generate potential full structures, and all results are downloadable in tsv format.

#### Additional databases

In 2018, LIPID MAPS added an In-Silico Structure Database (LMISSD). This is a relational database generated by computational expansion of headgroups and chains for a large number of commonly occurring lipid classes. LMISSD has been designed from an analytical chemistry perspective to enumerate all theoretically possible structures available from a large set of acyl/alkyl chains. A hierarchy of sum composition, chain composition and exact structures may be browsed for the various lipid classes. It contains over 1.1 million molecules, some 25 000 of which are in common with LMSD.

Ion Mobility spectrometry (IMS) provides an extra dimension to identifying lipids where gas-phase molecules are separated according to their interaction with a carrier gas generating a diagnostic collisional cross-section (CCS) value. To support those using this technique, an Ion Mobility database was developed in collaboration with the McLean research group (24). This contains experimental CCS values for lipid adducts searchable by lipid name and additionally linked from the individual lipid pages in LMSD.

### Database and curation infrastructure

LIPID MAPS databases were moved to an open-source platform about 10 years ago to ensure portability and long-term accessibility. The site is in the process of moving to the Laravel PHP framework. It currently runs on a CentOS Linux (release 7.9.2009) operating system (16GB RAM/8 CPU cores) using a Postgres (version 9.2.24) relational database. A Bingo (version 1.7.9) chemistry cartridge (25) is used to index lipid structures in molfile format and provide the capability to perform fast substructure searching as well as other functions such as molecular formula, mass and SMILES generation. LIPID MAPS uses the Javascript-based Ketcher (version 1.1) framework (www.lifescience.opensource.epam.com/ketcher/) to visualize structures within the browser. This is compatible with all common user platforms including mobile devices. Additional Javascript libraries allow users to calculate exact mass corresponding to different lipid adducts. Structure-related metadata such as physicochemical properties are computed during curation and displayed online for each lipid molecule. The entire LMSD database may be downloaded, with structures available in open-source molfile format, to facilitate re-use and incorporation into other cheminformatic workflows.

### Website search functionality

The search facility of the LIPID MAPS homepage was recently updated with an auto-complete functionality. The new search bar interrogates more database fields than previously, including InChIKey and identifiers from other databases, including ChEBI and KEGG (26). To aid searching using non-standard lipid names, RefMet (27) has been incorporated to standardize nomenclature enabling lipids to be found more easily. As an example, DAG C36:3 is converted to DG 36:3 prior to searching. Previously, if a numerical value was entered, the results returned were neutral masses from LMSD. To better support users, who generally search on *m/z* values from experimental data, the new search function operates differently. Instead, when users enter numeric values below 5000, they are redirected to the bulk search interface where they can choose specifically which form of mass value is most appropriate for their query. The cutoff of 5000 was chosen as values above this are unlikely to be lipid *m/z* values, but could be ChEBI IDs.

### Programmatic access

LIPID MAPS has implemented a REST API to provide programmatic access to data in LMSD and also LMPD (28). Recently, a SPARQL 1.1 endpoint (www.lipidmaps.org/sparql) was implemented as part of the ELIXIR 2021 ‘FAIR lipids’ Hackathon, so that users can run federated queries to other databases, such as SwissLipids (29) and UniProt (30). In addition to the lipids themselves, the lipids categories have been converted to RDF as owl:Classes using rdfs:subClassOf relations to reproduce the hierarchy.

### Tools

LIPID MAPS provides a variety of informatics tools to aid the lipidomics community as described previously (31). The structure drawing tools have been updated to include the additional class of phosphatidylthreonines. Tools to standardize lipid nomenclature, RefMet and LipidLynxX (32) have been incorporated into the site along with LipidFinder (33), a liquid chromatography/mass spectrometry workflow comprising peak filtering, MS searching and statistical analysis components. In addition, BioPAN, software developed to perform a pathway analysis from lipidomics datasets described elsewhere (22), is fully incorporated in the LIPID MAPS site.

A guide to bioinformatic software and tools for analysis of lipidomic data (34) has been produced in collaboration with EpiLipidNET and is available at www.lipidmaps.org/resources/tools?page=flow_chart.

### Education and outreach

LIPID MAPS has a strong focus on educational activities in lipidomics mass spectrometry, particularly supporting users of the database and resources. An online Spring School was hosted in 2020 at which 33 experts trained 485 early career researchers over five days. Since 2019, monthly webinars have been hosted with a total audience of 2.7K viewers. All these presentations are freely available on the LIPID MAPS YouTube channel (www.youtube.com/@lipidmaps3529), which has over 4800 h of content viewed. A podcast series of informal discussions has begun. The encyclopedic LipidWeb (www.lipidmaps.org/resources/lipid_web), created by Bill Christie is hosted under the LIPID MAPS banner and continues to be updated weekly. A weekly blog from Bill Christie was recently passed to Dan Raben for continuation (www.lipidmaps.org/updates/lipidmatters). LIPID MAPS partners with the EU COST Network EpilipidNET with their lipid community contributing to metabolic reactions and pathways, and novel lipid structures to LIPID MAPS databases.

### Future directions

Robust informatics resources supporting lipidomics are essential as we move into the emerging area of systems lipidomics, a subfield of systems biology which involves developing a holistic understanding of lipid behavior through the analysis of lipidomes, combined with corresponding data from other ‘-omics including proteomics and transcriptomics. Without high quality, expert curated databases, poorly interpreted data will be produced, leading to significant misutilization of time and resources. To address this, we aim to continue enriching data on discrete lipid structures, linking them to enzymes and genes via the biochemical reactions in which they participate.

Additional databases are needed to capture data on partly characterized lipid structure including modified lipids, the so-called epilipids (35) discovered in tissue samples along with their metadata, so that their study in disease can be facilitated. We plan to develop such resources in the future. Robust, standard practices for data analysis, and reporting is required (36) and LIPID MAPS is actively supporting the community to address these issues via our on-going training program in addition to database development and nomenclature.

## Data Availability

All LIPID MAPS databases (https://www.lipidmaps.org/) are licensed under a Creative Commons Attribution 4.0 International License.

## References

[1] Sud M. , Fahy E., Cotter D., Azam K., Vadivelu I., Burant C., Edison A., Fiehn O., Higashi R., Nair K.S. et al. Metabolomics Workbench: an international repository for metabolomics data and metadata, metabolite standards, protocols, tutorials and training, and analysis tools. Nucleic Acids Res. 2016; 44:D463–D70. 26467476 10.1093/nar/gkv1042 PMC4702780

[2] Fahy E. , Subramaniam S., Brown H.A., Glass C.K., Merrill A.H. Jr, Murphy R.C., Raetz C.R.H., Russell D.W., Seyama Y., Shaw W. et al. A comprehensive classification system for lipids. J. Lipid Res. 2005; 46:839–861. 15722563 10.1194/jlr.E400004-JLR200

[3] Sud M. , Fahy E., Cotter D., Brown A., Dennis E.A., Glass C.K., Merrill A.H. Jr, Murphy R.C., Raetz C.R.H., Russell D.W. et al. LMSD: LIPID MAPS structure database. Nucleic Acids Res. 2007; 35:D527–D32. 17098933 10.1093/nar/gkl838 PMC1669719

[4] Arita M. , Suwa K. Search extension transforms Wiki into a relational system: a case for flavonoid metabolite database. BioData Min. 2008; 1:7. 18822113 10.1186/1756-0381-1-7 PMC2556319

[5] Yabuzaki J. Carotenoids Database: structures, chemical fingerprints and distribution among organisms. Database. 2017; 2017:bax004. 28365725 10.1093/database/bax004 PMC5574413

[6] Artyukhin A.B. , Zhang Y.K., Akagi A.E., Panda O., Sternberg P.W., Schroeder F.C. Metabolomic ‘Dark Matter’ dependent on peroxisomal β-oxidation in Caenorhabditis elegans. J. Am. Chem. Soc. 2018; 140:2841–2852. 29401383 10.1021/jacs.7b11811 PMC5890438

[7] Fahy E. , Subramaniam S., Murphy R.C., Nishijima M., Raetz C.R.H., Shimizu T., Spener F., van Meer G., Wakelam M.J.O., Dennis E.A. Update of the LIPID MAPS comprehensive classification system for lipids. J. Lipid Res. 2009; 50:S9–S14. 19098281 10.1194/jlr.R800095-JLR200 PMC2674711

[8] Yore M.M. , Syed I., Moraes-Vieira P.M., Zhang T., Herman M.A., Homan E.A., Patel R.T., Lee J., Chen S., Peroni O.D. et al. Discovery of a class of endogenous mammalian lipids with anti-diabetic and anti-inflammatory effects. Cell. 2014; 159:318–332. 25303528 10.1016/j.cell.2014.09.035 PMC4260972

[9] Wilkinson M.D. , Dumontier M., Aalbersberg I.J.J., Appleton G., Axton M., Baak A., Blomberg N., Boiten J.-W., da Silva Santos L.B., Bourne P.E. et al. The FAIR Guiding Principles for scientific data management and stewardship. Sci. Data. 2016; 3:160018. 26978244 10.1038/sdata.2016.18 PMC4792175

[10] Juty N. , Le Novère N., Laibe C. Identifiers.org and MIRIAM Registry: community resources to provide persistent identification. Nucleic Acids Res. 2012; 40:D580–D6. 22140103 10.1093/nar/gkr1097 PMC3245029

[11] Liebisch G. , Fahy E., Aoki J., Dennis E.A., Durand T., Ejsing C.S., Fedorova M., Feussner I., Griffiths W.J., Köfeler H. et al. Update on LIPID MAPS classification, nomenclature, and shorthand notation for MS-derived lipid structures. J. Lipid Res. 2020; 61:1539–1555. 33037133 10.1194/jlr.S120001025 PMC7707175

[12] Schoch C.L. , Ciufo S., Domrachev M., Hotton C.L., Kannan S., Khovanskaya R., Leipe D., Mcveigh R., O’Neill K., Robbertse B. et al. NCBI Taxonomy: a comprehensive update on curation, resources and tools. Database. 2020; 2020:baaa062. 32761142 10.1093/database/baaa062 PMC7408187

[13] Europe PMC Consortium Europe PMC: a full-text literature database for the life sciences and platform for innovation. Nucleic Acids Res. 2015; 43:D1042–D1048. 25378340 10.1093/nar/gku1061 PMC4383902

[14] Martin E. , Monge A., Duret J.-A., Gualandi F., Peitsch M.C., Pospisil P. Building an R&D chemical registration system. J. Cheminform. 2012; 4:11. 22650418 10.1186/1758-2946-4-11 PMC3430593

[15] Hastings J. , Owen G., Dekker A., Ennis M., Kale N., Muthukrishnan V., Turner S., Swainston N., Mendes P., Steinbeck C. ChEBI in 2016: improved services and an expanding collection of metabolites. Nucleic Acids Res. 2016; 44:D1214–D1219. 26467479 10.1093/nar/gkv1031 PMC4702775

[16] Kim S. , Chen J., Cheng T., Gindulyte A., He J., He S., Li Q., Shoemaker B.A., Thiessen P.A., Yu B. et al. PubChem 2023 update. Nucleic Acids Res. 2023; 51:D1373–D1380. 36305812 10.1093/nar/gkac956 PMC9825602

[17] Chambers J. , Davies M., Gaulton A., Hersey A., Velankar S., Petryszak R., Hastings J., Bellis L., McGlinchey S., Overington J.P. UniChem: a unified chemical structure cross-referencing and identifier tracking system. Journal of Cheminformatics. 2013; 5:3. 23317286 10.1186/1758-2946-5-3 PMC3616875

[18] Armstrong D.R. , Berrisford J.M., Conroy M.J., Gutmanas A., Anyango S., Choudhary P., Clark A.R., Dana J.M., Deshpande M., Dunlop R. et al. PDBe: improved findability of macromolecular structure data in the PDB. Nucleic Acids Res. 2020; 48:D335–D343. 31691821 10.1093/nar/gkz990 PMC7145656

[19] Martens M. , Ammar A., Riutta A., Waagmeester A., Slenter D.N., Hanspers K., A Miller R., Digles D., Lopes E.N., Ehrhart F. et al. WikiPathways: connecting communities. Nucleic Acids Res. 2021; 49:D613–D621. 33211851 10.1093/nar/gkaa1024 PMC7779061

[20] Bansal P. , Morgat A., Axelsen K.B., Muthukrishnan V., Coudert E., Aimo L., Hyka-Nouspikel N., Gasteiger E., Kerhornou A., Neto T.B. et al. Rhea, the reaction knowledgebase in 2022. Nucleic Acids Res. 2022; 50:D693–D700. 34755880 10.1093/nar/gkab1016 PMC8728268

[21] Gillespie M. , Jassal B., Stephan R., Milacic M., Rothfels K., Senff-Ribeiro A., Griss J., Sevilla C., Matthews L., Gong C. et al. The reactome pathway knowledgebase 2022. Nucleic Acids Res. 2022; 50:D687–D692. 34788843 10.1093/nar/gkab1028 PMC8689983

[22] Gaud C. , C Sousa B., Nguyen A., Fedorova M., Ni Z., O’Donnell V.B., Wakelam M.J.O., Andrews S., Lopez-Clavijo A.F. BioPAN: a web-based tool to explore mammalian lipidome metabolic pathways on LIPID MAPS. F1000Res. 2021; 10:4. 33564392 10.12688/f1000research.28022.1 PMC7848852

[23] Liebisch G. , Vizcaíno J.A., Köfeler H., Trötzmüller M., Griffiths W.J., Schmitz G., Spener F., Wakelam M.J.O. Shorthand notation for lipid structures derived from mass spectrometry. J. Lipid Res. 2013; 54:1523–1530. 23549332 10.1194/jlr.M033506 PMC3646453

[24] Picache J.A. , Rose B.S., Balinski A., Leaptrot K.L., Sherrod S.D., May J.C., McLean J.A. Collision cross section compendium to annotate and predict multi-omic compound identities. Chem. Sci. 2019; 10:983–993. 30774892 10.1039/c8sc04396e PMC6349024

[25] Pavlov D. , Rybalkin M., Karulin B. Bingo from SciTouch LLC: chemistry cartridge for Oracle database. J. Cheminform. 2010; 2:F1.

[26] Kanehisa M. , Goto S. KEGG: kyoto encyclopedia of genes and genomes. Nucleic Acids Res. 2000; 28:27–30. 10592173 10.1093/nar/28.1.27 PMC102409

[27] Fahy E. , Subramaniam S. RefMet: a reference nomenclature for metabolomics. Nat. Methods. 2020; 17:1173–1174. 33199890 10.1038/s41592-020-01009-y

[28] Cotter D. , Maer A., Guda C., Saunders B., Subramaniam S. LMPD: LIPID MAPS proteome database. Nucleic Acids Res. 2006; 34:D507–D10. 16381922 10.1093/nar/gkj122 PMC1347484

[29] Aimo L. , Liechti R., Hyka-Nouspikel N., Niknejad A., Gleizes A., Götz L., Kuznetsov D., David F.P.A., van der Goot F.G., Riezman H. et al. The SwissLipids knowledgebase for lipid biology. Bioinformatics. 2015; 31:2860–2866. 25943471 10.1093/bioinformatics/btv285 PMC4547616

[30] Consortium U.P. UniProt: the Universal Protein Knowledgebase in 2023. Nucleic Acids Res. 2023; 51:D523–D531. 36408920 10.1093/nar/gkac1052 PMC9825514

[31] Fahy E. , Sud M., Cotter D., Subramaniam S. LIPID MAPS online tools for lipid research. Nucleic Acids Res. 2007; 35:W606–W612. 17584797 10.1093/nar/gkm324 PMC1933166

[32] Ni Z. , Fedorova M. LipidLynxX: a data transfer hub to support integration of large scale lipidomics datasets. 2020; bioRxiv doi:26 July 2020, preprint: not peer reviewed 10.1101/2020.04.09.033894.

[33] Fahy E. , Alvarez-Jarreta J., Brasher C.J., Nguyen A., Hawksworth J.I., Rodrigues P., Meckelmann S., Allen S.M., O’Donnell V.B LipidFinder on LIPID MAPS: peak filtering, MS searching and statistical analysis for lipidomics. Bioinformatics. 2019; 35:685–687. 30101336 10.1093/bioinformatics/bty679 PMC6378932

[34] Ni Z. , Wölk M., Jukes G., Mendivelso Espinosa K., Ahrends R., Aimo L., Alvarez-Jarreta J., Andrews S., Andrews R., Bridge A. et al. Guiding the choice of informatics software and tools for lipidomics research applications. Nat. Methods. 2022; 20:193–204. 36543939 10.1038/s41592-022-01710-0 PMC10263382

[35] Ni Z. , Goracci L., Cruciani G., Fedorova M. Computational solutions in redox lipidomics - current strategies and future perspectives. *Free**Radic*. Biol. Med. 2019; 144:110–123. 10.1016/j.freeradbiomed.2019.04.027 31035005

[36] McDonald J.G. , Ejsing C.S., Kopczynski D., Holčapek M., Aoki J., Arita M., Arita M., Baker E.S., Bertrand-Michel J., Bowden J.A. et al. Introducing the lipidomics minimal reporting checklist. Nat. Metab. 2022; 4:1086–1088. 35934691 10.1038/s42255-022-00628-3

